# Suction circuit flushing with chlorhexidine decreases ventilator-associated pneumonia: a quasi-experimental study

**DOI:** 10.3389/fmed.2023.1295277

**Published:** 2023-12-04

**Authors:** Mohamed H. Eid, Monica – Marilena Ţânţu, Jos M. Latour, Mohammed Ahmed Sultan, Nahed Attia Kandeel

**Affiliations:** ^1^School of Nursing and Midwifery, Faculty of Health, University of Plymouth, Plymouth, United Kingdom; ^2^Critical Care and Emergency Nursing Department, Faculty of Nursing, Mansoura University, Mansoura, Egypt; ^3^Medical Assistance and Physical Therapy Department, Faculty of Science, Physical Education and Informatics, University of Piteşti, Piteşti, Romania; ^4^Faculty of Nursing, Fudan University, Shanghai, China; ^5^Anaesthesia and Intensive Care Department, Faculty of Medicine, Mansoura University, Mansoura, Egypt

**Keywords:** airway management, chlorhexidine, endotracheal suctioning, intensive care units, ventilator-associated pneumonia, global health, green ICU

## Abstract

**Background:**

Endotracheal suctioning of mechanically ventilated patients differs across the world. In many low and middle-income countries, endotracheal suctioning is often performed with a sterile suctioning catheter that is used for 12 h or during the length of one nursing shift. The effect of flushing multiple used endotracheal suction system with chlorhexidine after suctioning to reduce ventilator associated pneumonia (VAP) remains unclear.

**Aim:**

The aim of the study is to assess the effectiveness of flushing multiple-used open endotracheal suction catheters and suctioning system with chlorhexidine gluconate 0.2% to reduce VAP in mechanically ventilated patients in a resource-limited Intensive Care Unit (ICU).

**Methods:**

Due to the difficulty of blinding the intervention for nurses who perform endo-tracheal suction procedures, we adopted a quasi-experimental method with a randomized controlled trial design. A sample of 136 ICU patients were allocated to the intervention (*n* = 68) or control group (*n* = 68) between May and November 2020. The intervention was flushing the multiple-used suction catheter and suction system with 40ml chlorhexidine gluconate 0.2% and in the control group we used normal saline to flush the catheter and suction system. The primary outcome was incidence of VAP and the cost of the flushing solutions was the secondary outcome measure.

**Results:**

Patients in the intervention group had a lower incidence of VAP compared to patients in the control group; 15 (22.1%) vs 29 (42.6%), *p* = 0.01. The incidence of late-onset VAP was 26.2% in the intervention group and 49% in the control group (*p* = 0.026) and the early-onset VAP was 13.2% in the intervention group and 25% in the control group (*p* = 0.081). Chlorhexidine gluconate 0.2% reduced the cost of suction system flushing (median: 78.4 vs 300 EGP, *p* < 0.001).

**Conclusion:**

Using chlorhexidine gluconate 0.2% to flush multiple-used suctioning catheters after every endo-tracheal suction procedure might reduce the incidence of VAP in mechanically ventilated patients. Chlorhexidine gluconate 0.2% can be a cost-effective solution for flushing the suction circuit. Nurses working in resource-limited ICUs and using suctioning catheters multiple times might consider using chlorhexidine gluconate 0.2% instead of normal saline or distilled water when flushing the suction system.

**Clinical trial registration:**

ClinicalTrials.gov, identifier NCT05206721.

## 1 Introduction

In low and middle-income countries, open endotracheal suction catheter is used multiple times to perform suctioning due to limited resources (1, 2). Currently, there is limited evidence for using a new suction catheter for each suction pass, acknowledged in a review article of endotracheal suction procedures in pediatric populations (3). Additionally, the latest artificial airway suctioning practice guidelines published by the American Association for Respiratory Care in 2022 did not mention any recommendations regarding suction catheter changing frequency (4). The guidelines adopted a study conducted in 2001 which showed that reusing an open tracheal suctioning catheter is safe and cost effective (5). Therefore, the current evidence of reusing suctioning catheters remains unclear, which rationalize the reason why some resource limited intensive care units (ICUs) using the catheter multiple times during a 12-h shift, and possibly explain the high ventilator associated pneumonia (VAP) incidence in these ICUs (1, 2).

Ventilator associated pneumonia is defined as pneumonia that develops 48 h or more after the initiation of mechanical ventilation in Intensive Care Unit (ICU) patients (6). The impact of VAP is recognized in the ICU as a main challenge because it prolongs the duration of mechanical ventilation, increases ICU length-of-stay and healthcare costs (7). The incidence of VAP has been reported to affect 5%–40% of patients receiving invasive mechanical ventilation for more than 2 days (8). The estimated attributable mortality of VAP is approximately 10%, with higher mortality rates among patients in surgical ICU (8).

Ventilator associated pneumonia is classified into two types according to the onset-time of pneumonia. Early-onset VAP occurs within the first 4 days of mechanical ventilation, whereas late-onset VAP occurs five or more days after mechanical ventilation initiation (9). Early-onset VAP is usually caused by community acquired pathogens, whereas late-onset VAP involves hospital flora (10). The most prevalent pathogens causing 80% of hospital acquired pneumonia are *Staphylococcus aureus*, *Klebsiella* species and *Pseudomonas aeruginosa* (11). Therefore, VAP is considered a hospital acquired condition and needs ongoing attention by ICU staff to prevent it.

Chlorhexidine is a well-known, widely used low-cost product, and is used as an antiseptic and disinfectant to kill microorganisms to reduce hospital-acquired infections spread in ICUs (12). It has been proposed as one of the five interventions in the care bundle to prevent VAP, daily oral care with chlorhexidine (13). Some studies have shown that intraoral application of chlorhexidine reduces VAP occurrence in mechanically ventilated patients (14–17). A trial reported that oral decontamination with 2% chlorhexidine concentration is more effective compared with 0.2% concentration in VAP prevention and oropharyngeal colonization reduction (18). We used 0.2% concentration to conduct this study because of its availability in the study setting. However, chlorhexidine is reported to be associated with some side effects including stinging, burning sensation of the tongue, reversible discoloration of the teeth and tongue, and transient disturbances of taste (19). Some studies recommended the exclusion of chlorhexidine from oral hygiene because of its side effects (20, 21). Our intervention used chlorhexidine gluconate 0.2% for flushing the suction system without patient’s integration to avoid any side effects and/or resistance.

Ventilator-dependent patients are at risk of increased secretions because of being sedated, and the presence of mechanical ventilator adjuncts which prevent spontaneous clearance of secretions (22). Therefore, endotracheal suctioning to clear secretions has been standard practice in the care of mechanically ventilated patients (23). It is recognized that the inner surface of artificial airways becomes colonized with biofilms containing pathogenic organisms and passing the suction catheter through it can dislodge these biofilms leading to inoculation of pathogenic organisms into the lungs, causing VAP (24). Therefore, Suctioning is a sterile procedure that is performed only when the patient needs without a routine schedule (25).

Suction catheter is used to remove tracheal secretions and may be either open or closed tracheal suctioning system (26). Some studies showed that there is no difference between using the open or the closed tracheal suctioning system method on the incidence of VAP or mortality rate (26–28). We used the open tracheal suctioning system to conduct this study as the closed system is not available in the study setting. Closed suction circuit catheters can be used multiple times, while the open tracheal suctioning catheter should be single use only (2, 3).

We decided to conduct this study for four reasons: (1) The endotracheal suction system might act as a good medium for the proliferation and colonization of pathogenic bacteria which can then migrate to patient’s lung during suctioning procedure causing VAP, which necessitate its cleaning and disinfection. (2) We have not identified in the literature any study investigating the effect of flushing this system with chlorhexidine gluconate 0.2% and whether it has an impact on VAP. (3) In low-middle income countries, many hospitals with resource limitations are still using one sterile suctioning catheter for 12 h or during a shift instead of single use, and the incidence of VAP is still high in these countries (1, 29). (4) Investigating whether chlorhexidine gluconate 0.2% will disinfect the multiple-used catheter to mimic the sterile single-used effect. Therefore, the aim of this study was to investigate the effect of flushing the open tracheal suctioning system with multiple-used catheters using chlorhexidine gluconate 0.2% on the occurrence of VAP among mechanically ventilated patients in limited resource intensive care units. We hypothesized that suction circuit flushing with chlorhexidine might reduce the incidence of VAP in mechanically ventilated patients compared to flushing with normal saline.

## 2 Materials and methods

A quasi-experimental design was adopted to conduct this study. Recruitment was between May and November 2020. During the study period, the recruitment was temporarily stopped for 3 months due to COVID-19 pressures. Ethical approval was obtained from the Local Research Ethical Committee (Ref. No. 245/2020). The study protocol was registered at ClinicalTrials.gov with the number NCT05206721.

### 2.1 Setting

This study was conducted at three ICUs located in one university hospital in Egypt. The three ICUs were a surgical, neurological and trauma respectively. Each ICU has 10 beds and provides care to mechanically ventilated patients. These units are well equipped with advanced technology required for high quality ICU care. The total admissions of the three ICUs are around 650 patients annually. The nurse-patient ratio in these units is 1 nurse to 2 patients. Standard VAP prophylaxis measures in these settings follow the ventilator bundle checklist published by Institute for Healthcare Improvement [IHI], 2012 including elevating head of the bed between 30 and 45 degrees, daily sedation vacation and readiness to weaning assessment, peptic ulcers disease prophylaxis, and deep venous thrombosis prophylaxis (30).

### 2.2 Participants

Patients admitted to the ICUs who were intubated within the previous 24 h and were expected to receive mechanical ventilation for more than 48 h were recruited into the study. The researchers used their clinical expertise and the patient ICU admission diagnoses to evaluate enrollment of the patients. Excluded were: (1) patients diagnosed with pneumonia at the time of admission and/or having a Modified Clinical Pulmonary Infection Score (MCPIS) of 5 or greater (31); (2) patients who had contraindications to suctioning (i.e., severe hemoptysis, increased intracranial pressure, and cerebrospinal fluid leaks); (3) patients with pulmonary edema, acute respiratory distress syndrome and atelectasis because of their pulmonary infiltrate disease pathophysiology; (4) patients known to be allergic to chlorhexidine.

### 2.3 Sample size and randomization

Based upon power analysis, the sample size was calculated using the free online software https://www.sphanalytics.com/sph-analytics-dss-research/and the values were set at 5% α error (95.0% significance) and 20.0% β error (80.0% power of the study). The sample size needed for the study was 136 patients. The patient admission number was used to randomly assign the patient to the intervention or control group. Patients with even admission numbers were assigned to the intervention group and the odd admission numbers to the control group (68 in each group).

### 2.4 Recruitment

Recruitment was performed by the Principal Investigator (PI). An initial assessment was performed on the first day for all mechanically ventilated patients using the MCPIS to confirm that they were free from pneumonia and exclusion criteria. Informed consent was obtained from the patients’ families (next of kin) who were informed about the aim, procedure, benefits, and risks of the study. The voluntariness nature of participation and the right to withdraw at any time without responsibility were also emphasized to them. Confidentiality of the participants’ personal information was maintained throughout the study procedure. Dropout patients before the 3rd day of admission were excluded and replaced by new cases to reach the sample target of 136 patients.

### 2.5 Intervention, standard care and procedures

The intervention group received chlorhexidine gluconate 0.2% as the flushing solution for the open tracheal suction system. The control group received normal saline as the flushing solution.

The PI did not develop a suctioning performance checklist according to standard guidelines or even ask staff nurses to modify their current suctioning practices neither in the study group nor in the control group. This was to ensure that VAP incidence was related to the use of chlorhexidine gluconate 0.2% as the flushing solution for the suction system effect and not a modified suctioning technique performed by the ICU nurses. Indication for suctioning in the study settings includes presence of secretions in patient’s chest and ausculation of crackles or wheezing patient’s chest.

#### 2.5.1 Intervention

The appropriate volume of chlorhexidine gluconate 0.2% solution required for effective flushing was investigated by the PI. The test of the total milliliter (ml) required chlorhexidine gluconate 0.2% for flushing the 1-meter open tracheal suction system with a catheter size of 16 Fr was 40ml. The details of the test are presented in Supplementary material I. The intervention as performed in four steps. Step I: Although allergic reactions to chlorhexidine are relatively rare, a sensitivity test was performed for the intervention group patients using chlorhexidine irrigation solution (0.1% concentration) for performing intradermal allergy test. Step II: Routine suctioning technique (following the hospital’s protocol) was performed on patients by nursing staff. Step III: The responsible nurse poured 40 ml chlorhexidine gluconate 0.2% solution from the chlorhexidine gluconate 0.2% bottle into a sterile container. Step IV: After suctioning the patient’s secretions, the nurse inserted the suction catheter into the 40 ml chlorhexidine gluconate 0.2% filled container and flushed the entire suction circuit with chlorhexidine. Before inserting the reused catheter, the nurse needed to do first 5 s of “dry suctioning” to make sure that there are no chlorhexidine gluconate 0.2% droplets in the catheter, to avoid chlorhexidine instillation into participants lungs.

#### 2.5.2 Standard care

The same procedure above was performed from step II to IV using normal saline (according to our standard protocol) instead of chlorhexidine gluconate 0.2% for flushing the open tracheal suction system; Routine suctioning technique (following the hospital’s protocol) was performed on patients by nursing staff. The responsible nurse poured 40 ml saline 0.9% from the normal saline bottle into a sterile container. After suctioning the patient’s secretions, the nurse inserted the suction catheter into the 40 ml saline filled container and flushed the entire suction circuit with normal saline. Critical care nurses performed hand washing, used sterile disposable gloves, kept the endotracheal suctioning catheter inside its plastic sheath after each suctioning procedure during the 12-h shift in both study groups.

### 2.6 Outcome measures

The occurrence of VAP was the primary outcome measure, and the cost of the flushing solutions was the secondary outcome measure. Regarding the primary outcome, the MCPIS was used to diagnose VAP and it was calculated on day three for early-onset VAP and on day six for late-onset VAP. The minimum score was 0, and the maximum score was 10. Patients who obtained scores above 5 were diagnosed with pneumonia, and those who scored below 5 were considered free of pneumonia. Patients who obtained a score of 5 (borderline) with hemodynamic stability, the PI re-evaluated these patients after 2 days (day eight). For hemodynamically unstable patients, the PI ordered a sputum culture (microbiological confirmation) on day six to verify their score. Patients with negative culture results obtained 0 points upon their score (MCPIS = 5) and were considered free of VAP, whereas those with positive culture obtained 2 more points upon their score (MCPIS = 7) and were considered to have VAP. Regarding the secondary outcome, the cost for each patient was calculated. the total number of required chlorhexidine gluconate 0.2% and saline bottles consumed by each patient in the intervention or control group was multiplied by their commercial price.

### 2.7 Data collection

Two data collection tools were used. The first tool, *data assessment tool*, was developed by the PI after reviewing relevant literature (16, 26, 32). The tool consists of three parts. The first part was the patients’ socio-demographics and health data, including age, gender, occupation, smoking habits, and health-relevant data such as date and reason of ICU admission, medical diagnosis, past medical history, ICU length-of-stay, and the modified Glasgow Coma Scale (MGCS) (32). The second part included mechanical ventilator modalities data, including the mechanical ventilation initiation date, artificial airway used, endotracheal tube size, mechanical ventilation mode and duration. The third part included the endotracheal suctioning data such as size of suction catheter, type of catheter connector, and duration of the total suctioning procedure (Supplementary material II).

The second data collection tool was the *VAP diagnostic criteria sheet* adopted from Singh et al. (31) and was used to assess the patients for clinical diagnosis of VAP. It includes the MCPIS based on five clinical assessments; each variable is worth 0–2 points including the patient’s body temperature, number of white blood cells, purulence and quantity of tracheal secretions (i.e., rare secretions, abundant, and purulent abundant secretions), oxygenation (calculated as PaO2 divided by the fraction of inspired oxygen [FiO2]), and chest radiography findings (no infiltrates, diffused infiltrates and localized infiltrates). The points for each variable of the MCPIS were summed, yielding a total score varied from 0 to 10 for data analysis (Supplementary material II).

The two data collection tools were tested before starting the data collection. The content validity of data assessment tool was assessed by seven experts in ICU nursing and medicine. The VAP diagnostic criteria sheet was adopted and has been extensively used in many studies. The data assessment tool and the VAP diagnostic criteria sheet were tested on 10% of the total sample (14 patients) to evaluate the tools’ clarity, feasibility, and applicability. Participants in the pilot study were excluded from the main study sample.

### 2.8 Statistical analysis

The obtained data were coded, computed, and statistically analyzed using IBM SPSS Statistics for Windows, version 24 (IBM Corp., Armonk, N.Y., USA). Data were presented as frequency and percentages (categorical variables) and mean, standard deviation (continuous variables). Distribution of data was assessed using the Shapiro–Wilk test and visual observation of histogram. Chi-square (χ2) was used for comparing categorical variables and was replaced with Fisher’s exact test or Monte Carlo exact test if the expected value of any cell was less than 5. Student’s *t*-test was used for comparing continuous variables. The median was used as a central tendency measure for continuous quantitative variables that were not normally distributed. The Mann–Whitney U-test (Z) was used to compare the two groups. The difference was considered significant at *p* ≤ 0.05.

## 3 Results

### 3.1 Patient’s sociodemographic and health-relevant data

**FIGURE 1:**
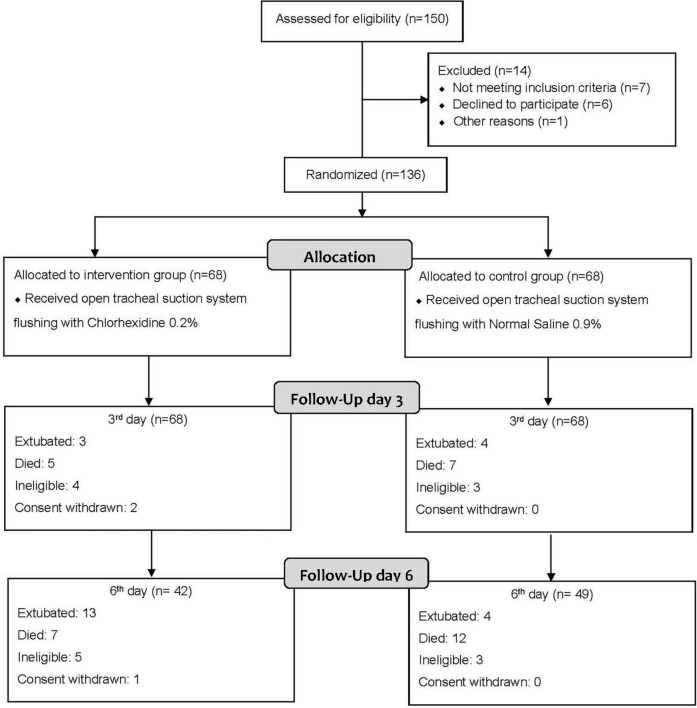
Flow diagram of study participants. All dropped out patients before the 3rd day have been excluded and replaced by new cases to fulfill our sample of 136 patients (68 in each group). While dropped out patients after the 3rd day and before day 6th were not replaced and have been evaluated for 3rd day early-onset VAP occurrence only.

In our study, 136 patients were enrolled of whom 68 patients were assigned to the intervention group. Patients lost in follow-up after the 3rd day and before day 6 were not replaced and have been evaluated for the early-onset VAP only (Figure 1).

There were no differences in age, severity of disease, underlying diseases between both groups, the majority of the study participants were male, and most patients were admitted for neurological disorders or trauma (Table 1). It was observed that the majority of patients had an ICU length-of-stay of more than 7 days. A statistically significant difference was observed in the mean modified GCS score between the two study groups on day 4 and 5 (Table 1).

**TABLE 1:** Sociodemographic and health relevant data of the study groups.

Variables	Intervention group	Control group	*p*-value
	***n* (%)**	***n* (%)**	
**Age in years**			
Mean (SD)	50.99 (20.61)	49.25 (17.23)	0.595
**Gender**			
• Male	49 (72.1%)	50 (73.5%)	0.847
• Female	19 (27.9%)	18 (26.5%)	
**Smoking habit**			
• Yes	18 (26.5%)	24 (35.3%)	0.265
• No	50 (73.5%)	44 (64.7%)	
**Reason for ICU admission**			
• Neurological disorders	40 (58.8%)	36 (52.9%)	0.116^1^
• Multiple trauma injuries	26 (38.2%)	22 (32.4%)	
• Cardiac disorders	0 (0%)	4 (5.8%)	
• ENT	1 (1.5%)	1 (1.5%)	
• Toxicology	1 (1.5%)	5 (7.4%)	
**Past medical history**			
• Yes	44 (64.7%)	42 (61.8%)	0.722
• No	24 (35.3%)	26 (38.2%)	
• Diabetes mellitus	26 (38.2%)	35 (52.9%)	0.121
• Hypertension	34 (50%)	32 (47.1%)	0.731
• Ischemic heart disease	18 (26.5%)	18 (26.5%)	−
• Renal failure	10 (14.7%)	8 (11.8%)	0.613
• Hepatic impairment	6 (8.8%)	4 (5.9%)	0.511
• Others	4 (5.9%)	3 (4.4%)	1.00
**Length of ICU stay (days)**			
• 3–4 days	15 (22%)	11 (16.1%)	0.445^1^
• 5–6 days	16 (23.5%)	22 (32.4%)
• ≥7 days	37 (54.5%)	35 (51.5%)
**Average MGCS [mean (SD)]**			
• Day 1	68 [7.09 (1.86)]	68 [7.32 (1.52)]	0.147
• Day 2	68 [6.54 (1.67)]	68 [6.92 (1.37)]	0.147
• Day 3	68 [6.22 (1.59)]	68 [6.73 (1.62)]	0.064
• Day 4	66 [5.91 (1.82)]	64 [6.78 (1.80)]	0.007*
• Day 5	48 [6.15 (1.67)]	55 [6.82 (1.78)]	0.052*
• Day 6	42 [6.090 (1.85)]	49 [6.38 (1.83)]	0.337

Ear, Nose, Throat diseases (ENT), Modified Glasgow Coma Score (MGCS). Data are presented as numbers (n), frequency (%), Mean and standard deviation [Mean (SD)]. *P* by Chi-Square test (χ^2^),

^1^Monte Carlo Exact Probability (MEP), *refers to significance if *P*-value ≤ 0.05. Others past medical history included (Asthma, Cancer, Epilepsy, and Stroke). It must also be clarified that after the 3rd day, we witnessed a daily drop out in the number of patients in the study groups due to patient’s recovery, MV weaning, unexpected ineligibility, sudden discharge/transfer, or death.

### 3.2 Ventilator modalities and endotracheal suctioning data

Most patients in the study groups were intubated via an endotracheal tube with a tube size of 7–7.5 mm and were on assisted modes of mechanical ventilation (Table 2). The duration of ventilation for most patients in both groups was ≥ 7 days. Concerning endotracheal suctioning data, no differences were observed between both groups (Table 2). We have not compared the fequency and duration of suctioning for each patient as it’s an individualized care procedure depends on presence of secretions for each participant.

**TABLE 2:** Ventilator modalities and endotracheal suctioning data of the study groups.

Variables	Intervention group	Control group	*p*-value
	***n* (%)**	***n* (%)**	
**Artificial airway used**			
• Endotracheal tube	67 (98.5%)	66 (97.1%)	1.00
• Tracheostomy tube	1 (1.5%)	2 (2.9%)	
**Intubation process**			
• Urgent	66 (97.1%)	65 (95.6%)	1.00^1^
• Elective	2 (2.9%)	3 (4.4%)	
**ETT size (mm)**			
• 5–5.5	1 (1.5%)	1 (1.5%)	0.071^1^
• 6–6.5	0 (0.00%)	4 (5.8%)	
• 7–7.5	49 (72.1%)	38 (55.9%)	
• 8–8.5	18 (26.4%)	25 (36.8%)	
**Mode of ventilation**			
• Controlled	5 (7.4%)	9 (13.2%)	0.126
• Assisted	60 (88.2%)	51 (75.0%)	
• Spontaneous	3 (4.4%)	8 (11.8%)	
**Duration of MV**			
• 3–4 days	21 (30.9%)	13 (19.1%)	0.184
• 5–6 days	13 (19.1%)	20 (29.4%)	
• ≥7 days	34 (50.0%)	35 (51.5%)	
**Suction catheter size (Fr)**			
• ≤10	1 (1.5%)	1 (1.5%)	0.278^1^
• 12	14 (20.6%)	17 (25.0%)	
• 14	46 (67.6%)	36 (52.9%)	
• 16	7 (10.3%)	14 (20.6%)	
**Type of SC connector**			
• Standard connector	42 (61.8%)	40 (58.8%)	0.861^1^
• Thumb control connector	19 (27.9%)	19 (27.9%)	
• Fingertip control connector	7 (10.3%)	9 (13.3%)	
**Duration of total suction time**			
• <30 s	8 (11.8%)	12 (17.7%)	0.298^1^
• 30–60 s	33 (48.5%)	37 (54.4%)	
• >1 min	27 (39.7%)	19 (27.9%)	

Endotracheal Tube (ETT), Mechanical Ventilation (MV), Millimeter (mm), Suction Catheter (SC), French gauge (Fr). Data are presented as numbers (n) and frequency (%), *P* by Chi-Square test (χ^2^),

^1^ Monte Carlo Exact Probability (MEP).

### 3.3 VAP incidence

The incidence of VAP among patients in the intervention group was lower than in the control group (22.1% vs 42.6%, *p* = 0.010). The in-depth focus of this incidence showed no statistically significant difference on the third day for early-onset VAP. However, statistically significant difference between both groups on day 6 for late-onset VAP incidence was observed (Table 3).

**TABLE 3:** Main study findings of the study groups.

Variables	Intervention group	Control group	*p*-value
	***n* (%)**	***n* (%)**	
**Total VAP**			
• Not VAP	53 (77.9%)	39 (57.4%)	0.010*
• VAP	15 (22.1%)	29 (42.6%)	
**Third day early-onset VAP**			
• Not VAP	59 (86.8%)	51 (75.0%)	0.081
• E-VAP	9 (13.2%)	17 (25.0%)	
**Sixth day late-onset VAP**			
• Not VAP	31 (73.8%)	25 (51.0%)	0.026*
• L-VAP	11 (26.2%)	24 (49.0%)	
**MCPIS [Mean (SD)]**			
• Day 1	68 [1.32 (1.11)]	68 [1.54 (1.15)]	0.258
• Day 3	68 [3.38 (1.39)]	68 [4.31 (1.20)]	0.001*
• Day 6	42 [4.24 (1.51)]	49 [5.04 (1.64)]	0.018*
• Day 8	2 [5.50 (2.12)]	5 [6.40 (2.70)]	0.696
Cost***			
• Mean (SD)	74.79 (30.14)	307.65 (115.49)	*p* < 0.001**
• Min–max	39.20 – 147.0	120.0 – 560.0	
• Median	78.4	300.0	

Ventilator associated pneumonia (VAP), Early-onset ventilator associated pneumonia (E-VAP), Late-onset ventilator associated pneumonia (L-VAP). Data are presented as numbers (n), frequency (%), Mean and standard deviation [Mean (SD)]. *P* by Chi-Square test (χ^2^), *refers to significance if *P*-value ≤ 0.05, **refers to high significance if *P*-value less than 0.001. ***Cost refers to Egyptian Pounds (EGP).

### 3.4 Economic cost

Statistically significant differences regarding the average cost of flushing solutions were observed between the two groups, with a significant cheaper commercial cost for chlorhexidine gluconate 0.2% compared to normal saline (Table 3).

## 4 Discussion

The aim of our study was to test a new intervention in endotracheal suction management in mechanically ventilated ICU patients. The intervention was to use chlorhexidine gluconate 0.2% in flushing the tracheal suction system, including the multiple use of a sterile suctioning catheter, compared to normal saline. We investigated the effect of this intervention on VAP incidence at day 3 and day 6 of ICU admission. The main findings of our study indicated that VAP incidence was reduced in the intervention group. Also, we identified that the cost of the chlorhexidine gluconate 0.2% was less than the standard care group. Also, we have not identified any adverse reactions in airways of study participants in the intervention group using chlorhexidine gluconate 0.2% during or after conducting the study.

To our knowledge and reviewing the current evidence, we have not identified any study describing a similar intervention. Therefore, we believe that this is the first study testing chlorhexidine gluconate 0.2% for flushing the suction system. Subsequently, rather than other studies comparing routine, our discussion is based on the analysis of the study results. Hopefully, our findings will shed light on a new knowledge gap, guiding further research for a more in-depth understanding of endotracheal suction procedures in resource-limited ICU.

Neurological disorders were the most common medical diagnosis among the study groups followed by multiple trauma injuries. This could be attributed to the fact that data were collected from surgical ICUs, which provide care to patients with surgical problems, neurological disorders, and those with multiple trauma injuries. In developing countries, neurological disorders contributed to 92 million disability-adjusted life-years in 2015 and were projected to 103 million in 2030 worldwide (33). In Egypt, neurological disorders are the leading cause of death accounting for 14.9% of total deaths (34).

The ICU length-of-stay in more than half of our study participants was more than 7 days without statistical differences. A prospective observational study, involving patients from 27 ICUs in nine European countries, reported that VAP resulted in 6% higher mortality, longer ICU length-of-stay (10 and 12 days), and increased duration of mechanical ventilation (35). Furthermore, systematic reviews with meta-analysis of randomized controlled trials revealed that VAP occurrence increased the ICU length-of-stay and duration of mechanical ventilation (36, 37). This might explain the reason why most of our study population with a confirmed VAP had an ICU length-of-stay of more than 7 days.

Concerning the average MGCS, statistically significant differences were noted between the two groups on days 4 and 5. Patients who developed VAP had a lower MGCS compared with non-VAP patients. Other investigations reported similar findings between patients who developed VAP and the non-VAP group regarding MGCS (38, 39).

According to our findings, it was noted that the majority of patients were intubated via an endotracheal tube sized 7–7.5 mm. Endotracheal intubation is a critical and life-saving intervention for airway management in ICUs (40). In an emergency, it is the first step to maintain a patent airway, allow adequate positive ventilation, and facilitate suctioning (41, 42). The standard adult tube size is 7.5–8 mm (24). Smaller tubes impede the clearance of secretions and create increased airflow resistance when weaning from the ventilator and might contribute to the development of VAP.

More than half of the participants underwent a suctioning procedure using the standard type of suction catheter connector. This is because it is the most widely known connector type in Egypt. In addition, we observed that it is the most preferable connector type for being not tedious, as suctioning initiation/stop can be controlled using one hand and the suction catheter insertion into the endotracheal tube is performed with the other hand. Moreover, slightly more than half of the participants consumed between 30 and 60 s as a total duration time for suctioning procedure. This is the usual time for suctioning procedure by the nursing staff in the study settings. However, it is widely recognized that suctioning duration should be limited to minimize adverse events. Additionally, it is recommended by American association of respiratory care, 2010 (43) that the duration of the suctioning event should be limited to less than 15 s. This highlights the need for training programs on suctioning for critical care nurses in the study settings.

The incidence of VAP among the patients in the intervention group was 22.1%, whereas the incidence among the control group patients was 42.6%. This indicates that using chlorhexidine gluconate 0.2% as a flushing solution might contribute to the reduction of VAP in ICU patients. These findings support other studies that reported the effectiveness of oral care or bed bathing using chlorhexidine solutions in reducing VAP incidence (12, 14, 17). One study conducted in Egypt investigated the effect of oral care with chlorhexidine on VAP incidence reported reduced VAP incidence (16). This might support our intervention to be applied with the care bundle to reduce the incidence of VAP.

The VAP bundle has been implemented in many ICUs, along with teamwork, and communication strategies (30). There is a level III evidence that successful implementation of care bundle results in decreased VAP rates (44). In Egypt, studies showed that implementation of VAP bundle in adult and neonatal ICU resulted in decreasing the incidence of VAP (45, 46). However, the bundle is not widely implemented in ICUs in Egypt. Therefore, we designed a new easy intervention to be implemented that might contribute to the reduction of VAP incidence as well as the costs of the flushing solution.

Based upon the day 3 early-onset VAP incidence in our study, no significant difference was noted between the study groups. This aligns with an Egyptian study that investigated the effects of oral care with chlorhexidine on VAP incidence and reported no statistically significant difference between the two groups regarding early-onset VAP incidence (16). However, our study demonstrated that on day 6 more patients in the control group were diagnosed with late-onset VAP, which was also confirmed in the Moustafa et al. study (16) reporting a significant difference between their study groups on day 6.

Finally, the average cost of chlorhexidine gluconate 0.2% used among the patients in the intervention group and the average cost (commercial price) of normal saline used among control group patients showed that chlorhexidine gluconate 0.2% suction system flushing intervention significantly decreased the cost of patient care.

Nurses’ performance and limited resources in many low and middle-income countries has direct contribution to the development of VAP (47). These countries need to adjust their own bundles with low-cost and high level of evidence variables adapted to the limited resources. Therefore, our intervention is a novel technique that has no harm and good impact on reducing the occurrence of VAP.

### 4.1 Limitations and recommendations

Our study warrants mentioning some limitations. Although our study used three ICU sites, these were all in one single hospital, which limits the generalizability of our study findings. Also, the sample size can be considered small and more large scale and multi-center studies are needed to confirm the effect of our intervention. Moreover, the VAP bundle and international suctioning guidelines were not rigorously implemented in the study settings. Another limitation is the blinding of the intervention. In a follow-up study we aim to blind the two solutions by giving the nurses the same shape container with different solution to limit the blinding. Also, the lack of uniformity in the training and gesture of bronchial breathing by nurses is another limitation to this study. Due to limited resources, the incidence of VAP was assessed using the MCPIS however the alveolar bronchoscopy is the accurate test for detecting VAP (diagnosis with BAL > 10^4^ CFU/ml). The study recruitment was temporarily stopped for 3 months due to COVID-19 pressures, and we are unsure this has affected the quality of the data collection. The duration of mechanical ventilation and the length of stay in the ICU should be reported using mean and standard deviation. Recording the frequency of suctioning procedures for each patient is necessary. A final limitation is the effect of the use of chlorhexidine on bacterial species. We did not perform additional lab testing to collect a sample from the suctioning collecting jar to identify bacterial species in the circuit after chlorhexidine flushing.

## 5 Conclusion

The findings of this study contribute to the evidence-based practice related to the care of ventilator-dependent patients. Endotracheal suction system flushing with chlorhexidine gluconate 0.2% might reduce the VAP incidence in ICU and reduce the cost of flushing solutions. Future large-scale studies on different patient populations and in different settings are also required to obtain solid evidence to support this approach and cover that significant gap of knowledge.

## Data availability statement

The raw data supporting the conclusions of this article will be made available by the authors upon reasonable request.

## Ethics statement

The studies involving humans were approved by the Research Ethics Committee of Faculty of Nursing, Mansoura University, Egypt. The studies were conducted in accordance with the local legislation and institutional requirements. The participants provided their written informed consent to participate in this study.

## Author contributions

MHE and NAK contributed to the design of the study. MHE, MAS, and NAK contributed to the data collection. MHE, MMŢ, and JML contributed to the data analysis and interpretation of data. MHE and JML drafted the first manuscript. MMŢ, MAS, and NAK provided revisions. All authors equally contributed, read, and approved the submitted version.
