# Supplementary material for: Suction circuit flushing with chlorhexidine decreases ventilator-associated pneumonia: a quasi-experimental study
Source: Front Med (Lausanne). 2023 Dec 4;10:1295277. doi: 10.3389/fmed.2023.1295277 (PMC10725984; doi:10.3389/fmed.2023.1295277)
Supplement: Supplementary file 1 [file Data_Sheet_1.docx]

Supplementary Material I

(Testing the required volume of chlorhexidine for flushing the suction system)

The appropriate amount of chlorhexidine gluconate 0.2% solution (manufactured by a well know Egyptian Company for Medical Supplies Manufacturing and Trading) required for effective suction system flushing was investigated by the PI through clinical trials under the supervision of clinical expertise (lecturer of Anesthesia and Critical Care Medicine, Master’s degree staff nurse) as follows:

- The PI performed a considerable endotracheal suctioning using the patient’s own suction catheter, after which the suction catheter was fully stacked of the patient’s secretions.
- The suction catheter was then primed with a considerable amount of methylene blue diluted in normal saline (1:200) which gave a blue color to the suctioning catheter, tube, and jar (Figure 1).


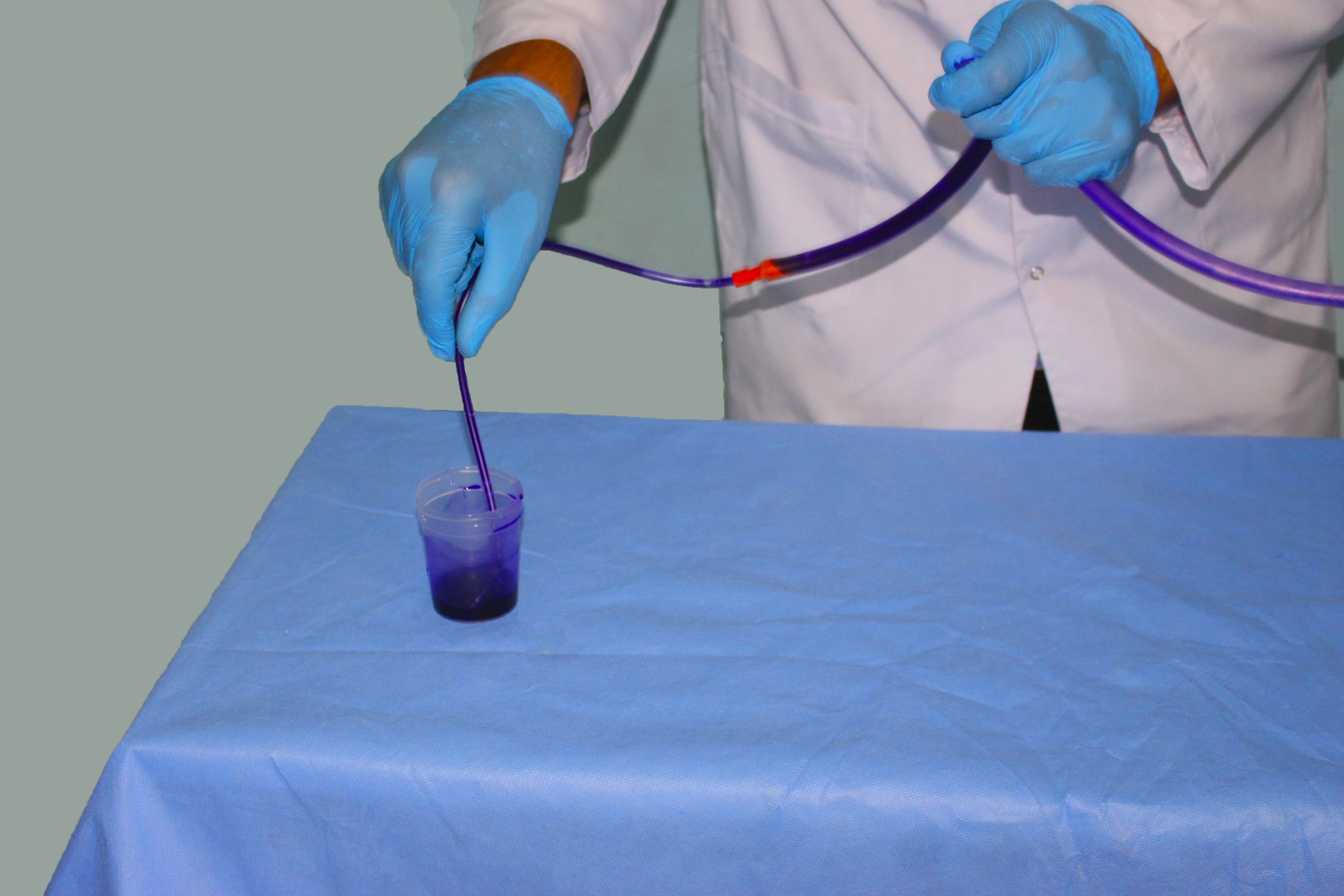


***Figure 1.*** Priming the Suctioning Circuit with Methylene Blue

- Flushing trials started with an amount of 10 ml, 20 ml, 30 ml, etc. of chlorhexidine solution to check the appropriate amount for removing the methylene blue color and cleaning the suction circuit from any stacked secretions (Figures 2, 3 & 4).


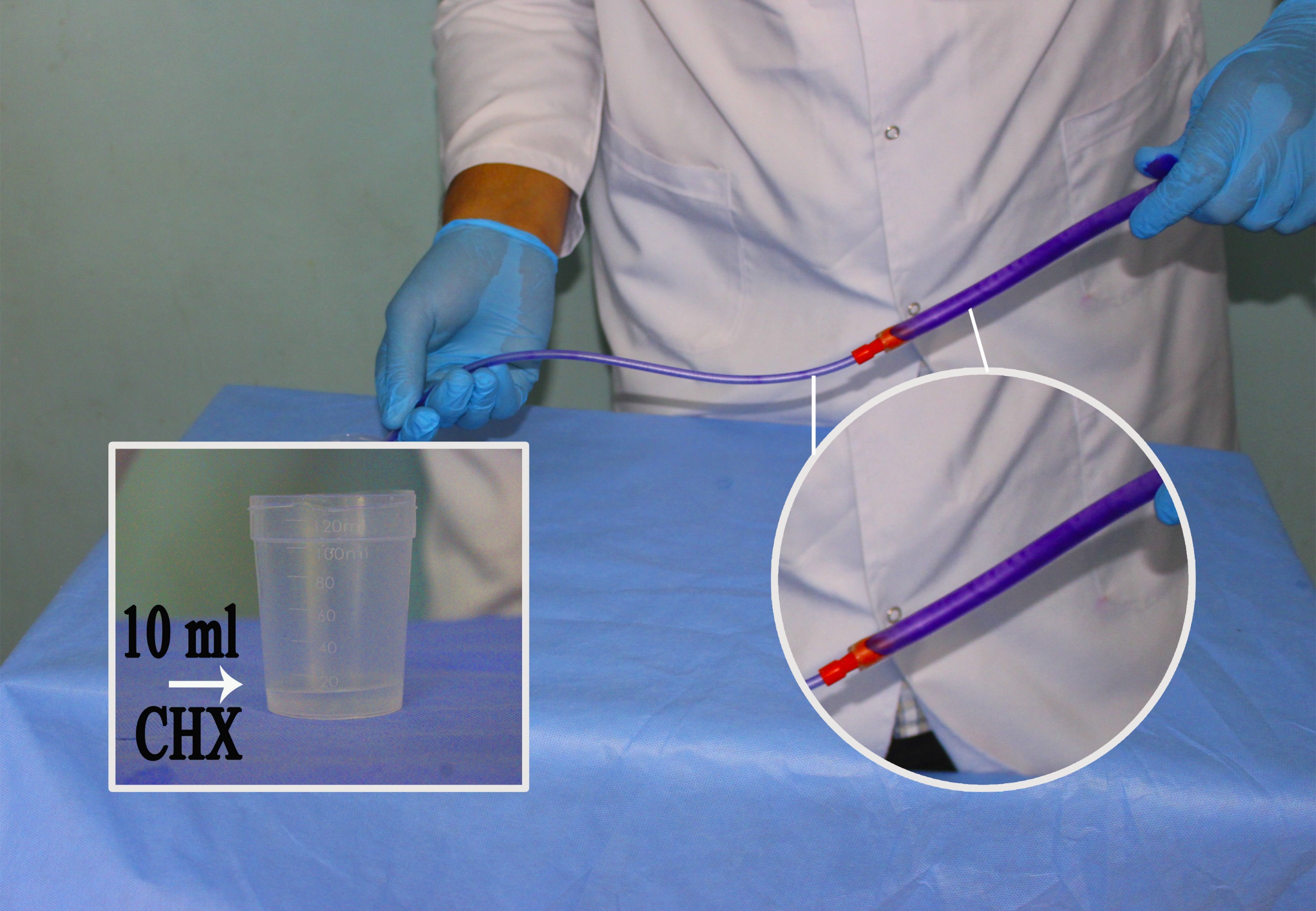


***Figure 2.*** The Clearance Effect of 10 ml Chlorhexidine Flushing


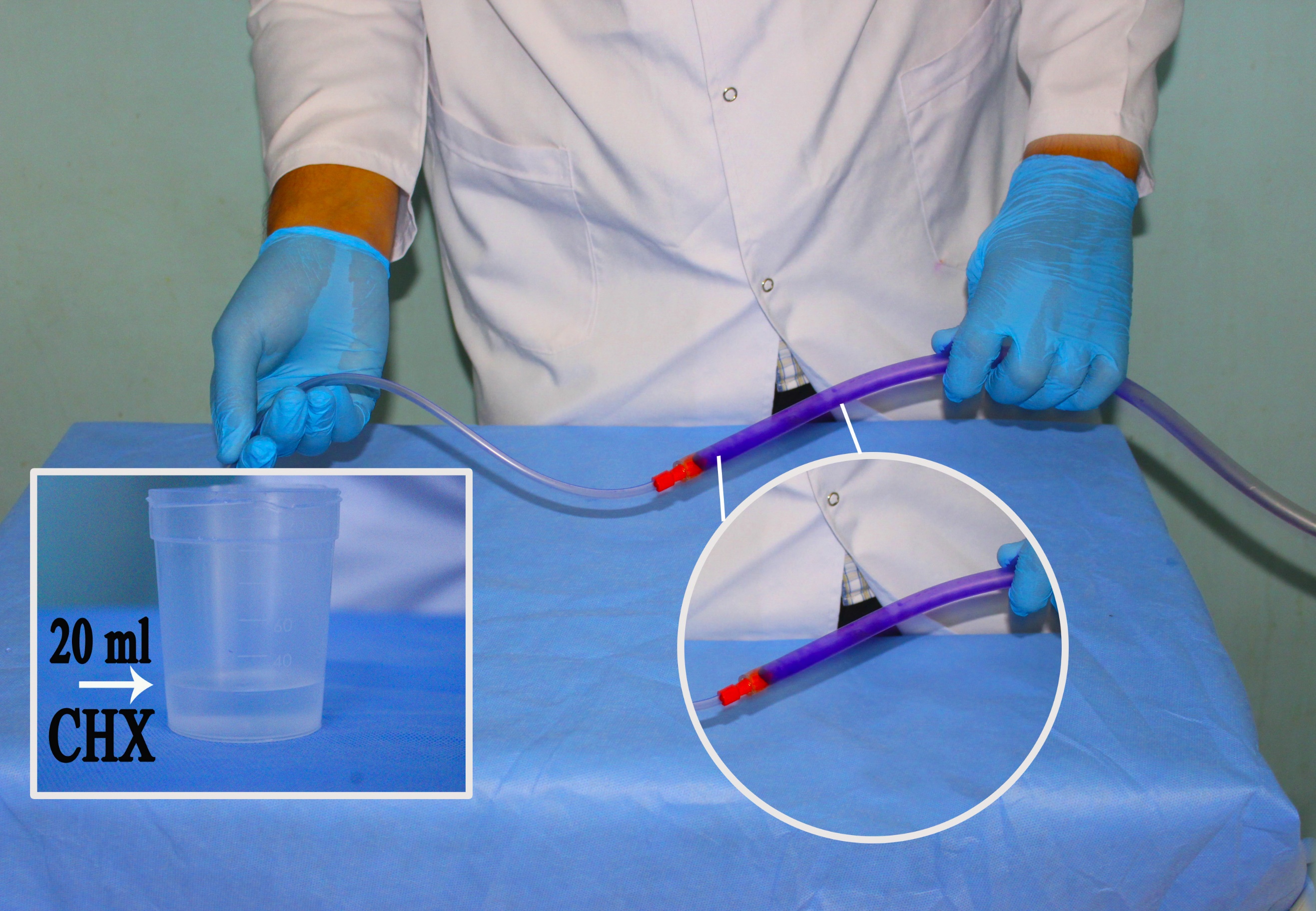


***Figure 3.*** The Clearance Effect of 20 ml Chlorhexidine Flushing


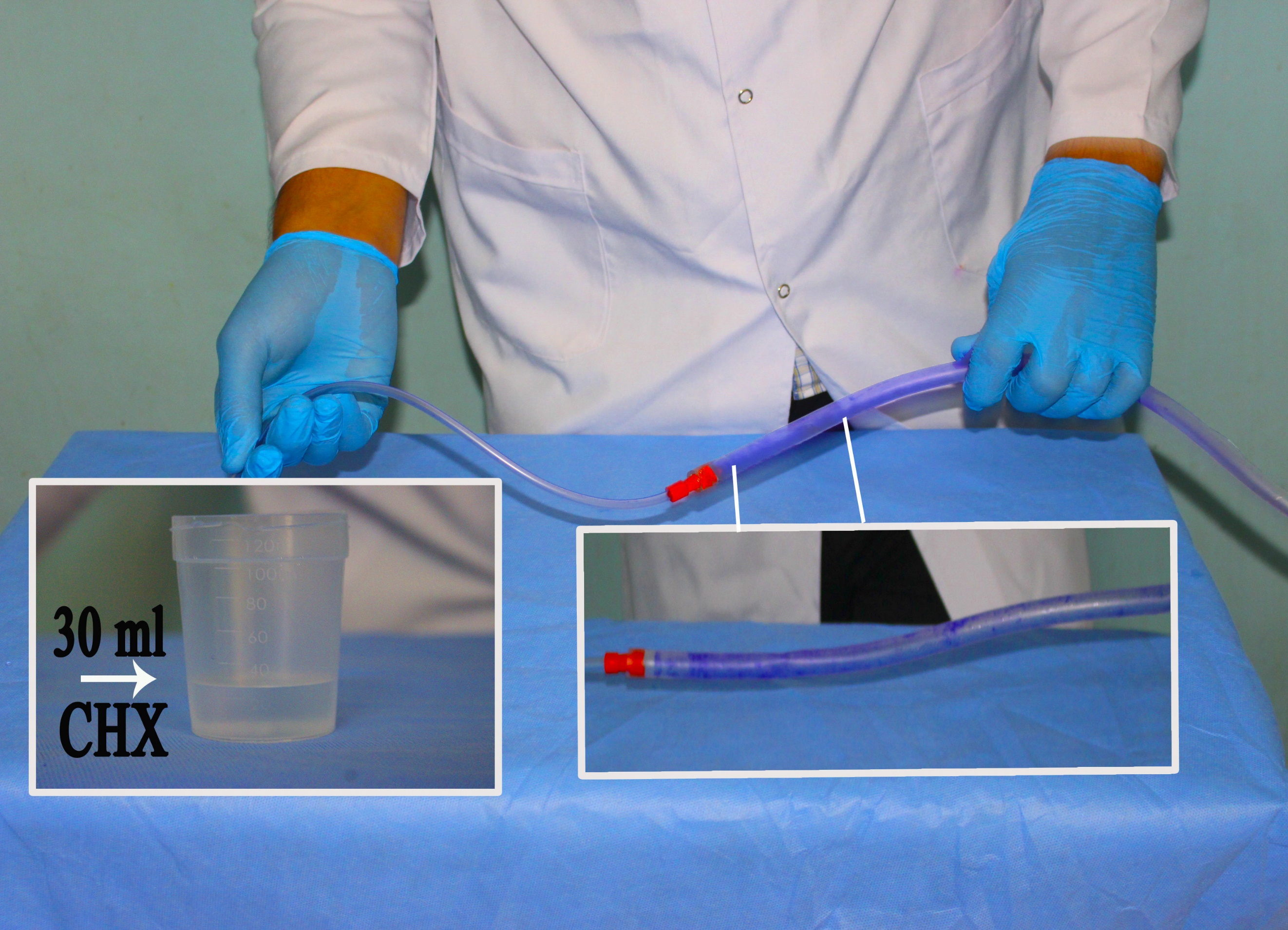


***Figure 4.*** The Clearance Effect of 30 ml Chlorhexidine Flushing

- The results showed that 40 ml of chlorhexidine solution was an adequate amount for suction circuit flushing and removal of any stacked secretions in the suction tube of
  1-meter length and catheter size of 16 Fr (Figure 5).


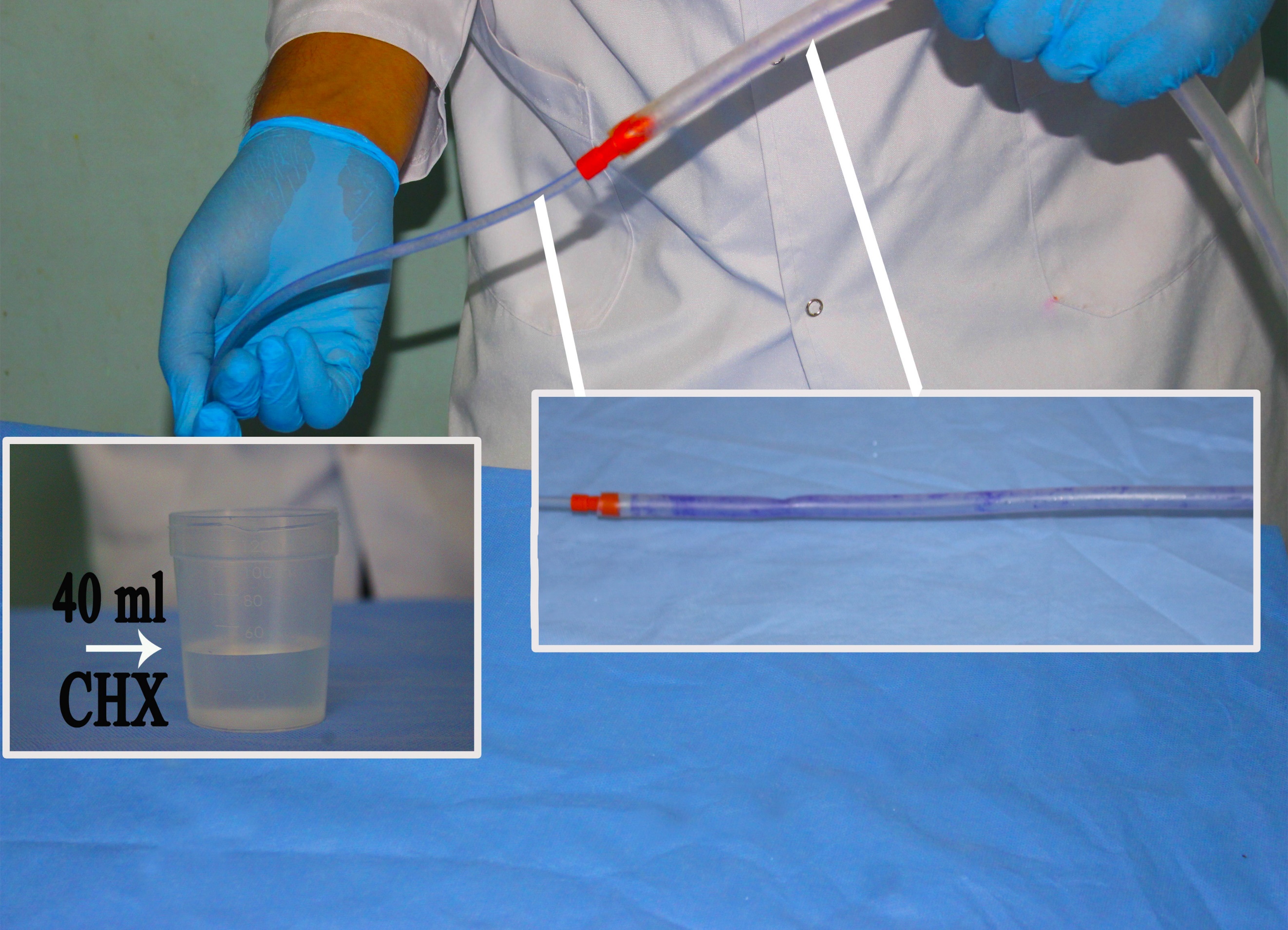


***Figure 5.*** The Clearance Effect of 40 ml Chlorhexidine Flushing
