# Supplementary material for: Suction circuit flushing with chlorhexidine decreases ventilator-associated pneumonia: a quasi-experimental study
Source: Front Med (Lausanne). 2023 Dec 4;10:1295277. doi: 10.3389/fmed.2023.1295277 (PMC10725984; doi:10.3389/fmed.2023.1295277)
Supplement: Supplementary file 2 [file Data_Sheet_2.docx]

Supplementary Material II

**(Data Collection Tool)**

**List of Abbreviations**

| **Abbreviation** | **Full name** |
| --- | --- |
| **GCS** | Glasgow Coma Score |
| **ICU** | Intensive Care Unit |
| **CSSF** | Chlorhexidine Suction System Flushing |
| **SSSF** | Saline Suction System Flushing |

**Open Suction Circuit Flushing with Chlorhexidine decreases Ventilator-Associated Pneumonia: A Quasi-experimental study**

- **Patient's Name:**

**Patient's Code:**

- **Admission Number:**
- **Study Group:**

| **CSSF** |  |
| --- | --- |
| **SSSF** |  |

**Tool I: Data Assessment Tool**

**Part 1: Patient's Sociodemographic and Health Relevant Data**

1. **Patient's Characteristics:**

| 1. **Age:**   10-20  31-40  51-60 |  | 21- 30  41-50  Above 60 |  |
| --- | --- | --- | --- |
| 1. **Gender** |  |  |  |
| Male |  | Female |  |
| 1. **Occupation:** |  |  |  |
| Employee |  | Farmer/Worker |  |
| Housewife |  | Retired |  |
| Others (specify): |  |  |  |
| 1. **Educational Level:** |  |  |  |
| Illiterate |  | Primary |  |
| Preparatory |  | Secondary |  |
| Bachelor |  | Others |  |
| 1. **Smoking Habits:** |  |  |  |
| Yes |  | No |  |

1. **Health Relevant Data:**

| 1. **Date of admission to ICU: / /** | |
| --- | --- |
| 1. **Reason for admission:** | |
| Respiratory Failure | Multiple Injury |
| Cardiac Disease | Renal Disease |
| Neurological Disease | Others Specify……………… |
| 1. **Medical diagnosis:** |  |
| 1. **Past medical history:** |  |
| Diabetes Mellitus | Hypertension |
| Ischemic heart disease | Renal failure |
| Hepatic impairment | Others specify……………… |

1. **Duration of ICU Stay**

| 1-2 days | 3-4 days |
| --- | --- |
| 5-6 days | ≥7 days |

1. **Level of Consciousness Based on Glasgow Coma Scale (GCS):**
2. **On admission:**
3. **Daily assessment:**

| Day/shift | 1^st^ day | 2^nd^ day | 3^rd^ day | 4^th^ day | 5^th^ day | 6^th^ day |
| --- | --- | --- | --- | --- | --- | --- |
| GCS |  |  |  |  |  |  |

**Part II: Ventilator Modalities Data**

1. **Mechanical Ventilation Initiation Date:**
2. **Artificial airway**

| Endotracheal Tube | Tracheostomy |
| --- | --- |
| Laryngeal Mask | Others |

1. **Intubation Process**

| Urgent | Elective |
| --- | --- |

1. **Size of Endotracheal Tube**

| 5-5.5 | 6-6.5 |
| --- | --- |
| 7-7.5 | 8-8.5 |

1. **Mode of Ventilation**

| Controlled |
| --- |
| Assisted |
| Spontaneous |

1. **Duration of Mechanical Ventilation**

| 1-2 days | 3-4 days |
| --- | --- |
| 5-6 days | ≥7 days |

**Part III: Endotracheal Suctioning Data**

| 1. **Size of Suction Catheter** |  |
| --- | --- |
| ≤10 fr | 12 fr |
| 14 fr | 16 fr |
| 18 fr | 20 fr |
| 1. **Type of Suction Catheter Connector** | |
| Standard Connector |  |
| Thumb Control Connector |  |
| Fingertip Control Connector |  |
| 1. **Duration of Total Suction Time** |  |
| ˂ 30 Seconds |  |
| 30 Seconds -1 Minute |  |
| ˃ 1 Minute |  |

**Tool ΙΙ: VAP Diagnostic Criteria Sheet**

**The Modified Clinical Pulmonary Infection Score (MCPIS)**

| **MCPIS Elements** | **Range** | **Score** | **Day 1** | **Day 3** | **Day 6** |
| --- | --- | --- | --- | --- | --- |
| **Temperature** | ≥ 36.5 and ≤ 38.4 | 0 |  |  |  |
|  | ≥ 38.5 and ≤ 38.9 | 1 |  |  |  |
|  | ≥ 39 and ≤ 36 | 2 |  |  |  |
| **Blood Leukocytes Count, per mm^3^** | 4,000 – 11,000 | 0 |  |  |  |
|  | ˂4,000 or ˃11,000 | 1 |  |  |  |
|  | ˂4,000 – ˃11,000 + band forms ≥ 500 | 2 |  |  |  |
| **Tracheal Secretions** | Rare | 0 |  |  |  |
|  | Abundant | 1 |  |  |  |
|  | Abundant + Purulent | 2 |  |  |  |
| **Oxygenation PaO_2_/FiO_2_** | ˃ 240 or ARDS | 0 |  |  |  |
|  | ≤ 240 and no evidence of ARDS | 2 |  |  |  |
| **Chest X-ray Infiltrates** | No infiltrates | 0 |  |  |  |
|  | Diffused | 1 |  |  |  |
|  | localized | 2 |  |  |  |
| **Total Score** | **˃ 5 = VAP** |  |  |  |  |
